# The Synthesis of Pentyl Leaf Volatiles and Their Role in Resistance to Anthracnose Leaf Blight

**DOI:** 10.3389/fpls.2021.719587

**Published:** 2021-08-26

**Authors:** Zachary Gorman, Jordan P. Tolley, Hisashi Koiwa, Michael V. Kolomiets

**Affiliations:** ^1^Department of Plant Pathology and Microbiology, Texas A&M University, College Station, TX, United States; ^2^Department of Horticultural Sciences, Texas A&M University, College Station, TX, United States

**Keywords:** green leaf volatile (GLV), volatile organic compound (VOC), *Colletotrichum graminicola*, lipoxygenase (LOX), priming, oxylipin, ketol

## Abstract

Volatiles are important airborne chemical messengers that facilitate plant adaptation to a variety of environmental challenges. Lipoxygenases (LOXs) produce a bouquet of non-volatile and volatile oxylipins, including C_6_ green leaf volatiles (GLVs), which are involved in a litany of plant physiological processes. GLVs are emitted by a diverse array of plant species, and are the best-known group of LOX-derived volatiles. Five-carbon pentyl leaf volatiles (PLVs) represent another widely emitted group of LOX-derived volatiles that share structural similarity to GLVs, however, relatively little is known about their biosynthesis or biological activity. In this study, we utilized PLV-deficient mutants of maize and Arabidopsis and exogenous PLV applications to elucidate the biosynthetic order of individual PLVs. We further measured PLVs and GLVs after tissue disruption of leaves by two popular methods of volatile elicitation, wounding and freeze-thawing. Freeze-thawing distorted the volatile metabolism of both GLVs and PLVs relative to wounding, though this distortion differed between the two groups of volatiles. These results suggest that despite the structural similarity of these two volatile groups, they are differentially metabolized. Collectively, these results have allowed us to produce the most robust PLV pathway to date. To better elucidate the biological activity of PLVs, we show that PLVs induce maize resistance to the anthracnose pathogen, *Colletotrichum graminicola*, the effect opposite to that conferred by GLVs. Further analysis of PLV-treated and infected maize leaves revealed that PLV-mediated resistance is associated with early increases of oxylipin α- and γ-ketols, and later increases of oxylipin ketotrienes, hydroxytrienes, and trihydroxydienes. Ultimately, this study has produced the most up-to-date pathway for PLV synthesis, and reveals that PLVs can facilitate pathogen resistance through induction of select oxylipins.

## Introduction

Volatile-mediated signaling is an important aspect of plant life that allows plants to endure a plethora of environmental challenges. Plant volatile signaling facilitates inter- and intra-plant communication, as well as communication with insects and microbes (Allmann and Baldwin, 2010; Matsui et al., 2012). These communications allow plants to anticipate and pre-emptively prime defenses against a wide range of imminent stresses (Engelberth et al., 2004). There are several key groups of volatile organic compounds (VOC) that are integral to these processes, including green leaf volatiles (GLV); a group of six-carbon, lipid-derived, volatile oxylipins that are synthesized in the hydroperoxide lyase (HPL) branch of the lipoxygenase (LOX) pathway (Matsui, 2006). These volatiles are widely emitted in response to a variety of different stresses and can induce widespread defense (Ameye et al., 2018). However, as our recent work showed, GLVs can also contribute to susceptibility of maize to the fungal agent of anthracnose disease, *Colletotrichum graminicola* (Gorman et al., 2020). While GLVs are a major component of VOC blends, they are not the only group of LOX-derived VOCs. Select jasmonates (Loughrin et al., 1995; Seo et al., 2001), and lesser-known five-carbon volatiles, henceforth referred to as pentyl leaf volatiles (PLVs), are also widely emitted LOX-derived volatile oxylipins.

Pentyl leaf volatiles are a group of volatiles that consist of five-carbon aldehydes, alcohols, ketones, and acetate conjugates produced in the LOX branch of the LOX pathway. Like GLVs, PLVs are emitted in response to a litany of abiotic and biotic stresses in numerous plant species (Fall et al., 2001; Heiden et al., 2003; Jardine et al., 2012; Mochizuki et al., 2016). Despite this, there has been little prior focus on the physiological functions of these volatiles or their synthesis. However, important roles of these volatiles in various plant species have been previously suggested, including priming of plant defenses against pathogens (Alméras et al., 2003; Song and Ryu, 2013; Choi et al., 2014; Song et al., 2015), aphid defense (Song and Ryu, 2013), and attraction of insects (van Tol et al., 2012; Song and Ryu, 2013; Roberts et al., 2019). Though PLVs include a diverse array of individual molecular species, little is known about the order of their synthesis.

**FIGURE 1:**
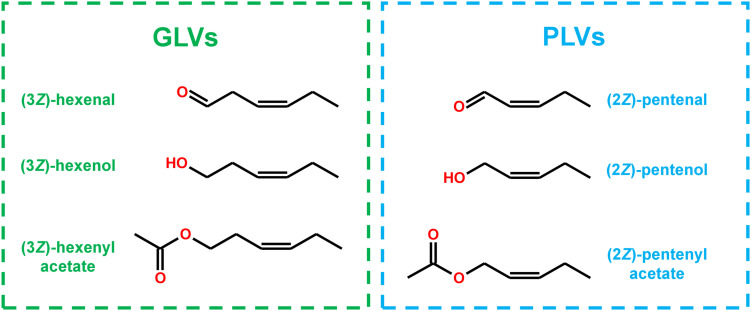
Structural similarity of GLVs and PLVs.

Structurally, many PLV molecular species display distinct similarity to GLV molecular species (Figure 1), and are often co-emitted in response to stress (Fall et al., 2001; Heiden et al., 2003; Jardine et al., 2012; He et al., 2020). Both of these VOC groups depend on utilization of 13*S*-hydroperoxy octadecatrienoic acid (13*S*-HPOT) and 13*S*-hydroperoxy octadecadienoic acid (13*S*-HPOD), which are generated by LOX-mediated oxygenation of linolenic (C18:3) and linoleic acids (C18:2), respectively (Salch et al., 1995; Matsui, 2006). HPL acts on these hydroperoxides to produce the primary GLVs, (3*Z*)-hexenal and hexanal (Mukhtarova et al., 2018), as well as a non-volatile 12-carbon compound, (9*Z*)-traumatin (Hatanaka, 1993). (3*Z*)-Hexenal can be enzymatically or non-enzymatically isomerized to another aldehyde, (2*E*)-hexenal (Hatanaka, 1993; Kunishima et al., 2016; Spyropoulou et al., 2017), and both of these aldehydes can be enzymatically reduced to corresponding alcohols by NADH-dependent alcohol dehydrogenases (ADH) (Bate et al., 1998; Speirs et al., 1998) and NADPH-dependent cinnamaldehyde and hexenal reductase (CHR) (Tanaka et al., 2018). These alcohols can then be further converted to their respective acetate-conjugates by acetyl-CoA-dependent BAHD acetyl transferases (AT) (D’Auria et al., 2007).

Though LOXs oxygenate polyunsaturated fatty acids, they may also perform a secondary reaction on LOX-derived 13*S*-HPOT. This generates a radical that undergoes spontaneous β-scission to form a pentene allylic radical that rapidly forms the isomers, 1-penten-3-ol and (2*Z*)-pentenol. Pentanol may also be produced from 13*S*-HPOD substrate, although LOXs seem to have less affinity for this substrate (Salch et al., 1995). LOX-mediated cleavage of 13-hydroperoxides also generates a non-volatile 13-carbon compound, 13-oxo-9(*Z*)-11(*E*)-tridecadienoic acid (OTD) (Vliegenthart et al., 1975, 1977; Salch et al., 1995; Gao et al., 2008; He et al., 2020). (2*Z*)-Pentenol and 1-penten-3-ol can be metabolized into aldehydes or ketones, respectively, through the activity of ADHs (Gardner et al., 1996). Beyond this, little is known about derivatization of these volatiles, each of which may have their own distinct signaling activity. Since both GLVs and PLVs share common substrate, it is not surprising that the major PLV-producing LOX isoforms identified in maize, ZmLOX10 (He et al., 2020), Arabidopsis, AtLOX2 (Mochizuki et al., 2016), and tomato, TomLOXC (Shen et al., 2014) are also the sole GLV-producing LOX isoforms in these respective species (Christensen et al., 2013). Notably, ZmLOX6 of maize lacks the ability to generate fatty acid hydroperoxides, but can cleave them into PLVs and OTD (Gao et al., 2008). Soybean also appears to have two LOX isoforms that contribute to PLV synthesis (Kobayashi et al., 1995; Fisher et al., 2003).

Utilizing PLV-deficient mutants of maize and Arabidopsis alongside exogenous application of PLVs, we establish the biosynthetic order of PLVs in the LOX pathway and provide evidence that despite the similar structure of PLVs and GLVs, they are metabolized differently. We also provide evidence that freeze-thawing of plant tissues, a popular method of volatile analysis, distorts the volatile oxylipin profiles of plant tissues, and differentially effects metabolism of PLVs and GLVs. Lastly, we show that in maize, PLVs induce resistance to *Colletotrichum graminicola*, which stands in contrast to results of our recent study that revealed GLVs promote disease progression to this pathogen (Gorman et al., 2020). Furthermore, we show that PLV-mediated resistance correlates with the synthesis of oxylipin α- and γ-ketols, ketotrienes, hydroxytrienes, and trihydroxydienes.

## Materials and Methods

### Plant and Fungal Materials and Growth Conditions

Mutant alleles of ZmLOX10 in maize were obtained by PCR screening of the *Mutator*-transposon insertional genetics resource at Corteva Agriscience, formerly DuPont-Pioneer^1^, for insertions in these genes. The *lox10-3* mutant allele was confirmed as an exon-insertional knockout mutant (Christensen et al., 2013). Original *lox10-3* mutants were backcrossed into the B73 and W438 inbred line backgrounds and genetically advanced to the backcross 7 stage. Maize plants used in all experiments were grown to the V4 stage (plants having four fully expanded leaves) under growth lights (∼300 μmol m^–2^ s^–1^) in a 14:10 h (light:dark) regime at 21–24°C. Maize plants were grown in TX-360 Metro Mix soil (Sun Gro Horticulture, Agawam, MA, United States).

*Arabidopsis thaliana* Col-0 and *Atlox2-1* mutants in the Col-0 background were grown for 4 weeks in LP5 potting medium (Sun Gro Horticulture, Bellevue, WA, United States) in a growth chamber (∼85 μmol m^–2^ s^–1^) under a 12:12 h (light:dark) regime at 23°C (light): 21°C (dark), in 65% relative humidity. Screening of *Atlox2-1* mutant lines was performed as previously described by Glauser et al. (2009).

All *C. graminicola* plates used in infection assays were grown from culture stock (*C. graminicola* 1.001 strain) kept in a –80°C freezer. Cultures were grown on PDA plates for at least 2 weeks before conidia were collected for use in plant inoculations. Spore extractions were performed as previously described by Gao et al. (2007) and were used within 2 h of extraction.

### PLV Metabolism Assays

For maize, fully expanded third leaves of wild type (WT) and *lox10-3* mutant in the B73 background were enclosed in 800 mL jars (one leaf per jar) alongside a cotton ball containing 2.5 μL of 100 mM of select PLVs dissolved in dichloromethane. For Arabidopsis, intact WT or *Atlox2-1* mutants were grown in small pots (∼350 mL) (5 plants/pot), were enclosed in these jars with the respective treatments. Col-0 was exposed to 5 μL of treatments, *Atlox2-1* mutants were exposed to 2.5 μL. Four replicates for each plant/genotype/treatment were used. PLV treatments included purified chemical standards of (2*Z*)-pentenol, (2*E*)-pentenol, (2*E*)-pentenal, pentanol, pentanal, 1-penten-3-ol, 1-penten-3-one, 3-pentanol, and 3-pentanone (Sigma-Aldrich, St. Louis, MO, United States). Plants were incubated alongside the PLV treatments for 20 min, and then volatiles were collected onto HaySepQ filters containing 80–100 mesh (Supelco, Bellefonte, PA, United States) via dynamic airflow (1 L min^–1^) for an additional 20 min. Volatiles were eluted off the HaySepQ filter traps with 250 μL of dichloromethane containing 100 μM of the internal standard, (4*Z*)-hexenol (Sigma-Aldrich, St. Louis, MO, United States). Volatiles were analyzed and quantified via gas chromatography mass spectrometry (GC-MS).

### Volatile Analysis of Wounding and Freeze-Thawing

For volatile analysis of wounding responses, leaves of both maize and Arabidopsis were excised, quickly weighed out to 2 g, and cut into 1 cm pieces that were immediately placed into 800 mL jars (He et al., 2020). For maize, the 3rd and 4th leaves of B73, W438, and *lox10-3* mutants in these backgrounds were used (one plant/replicate, four replicates total). For Arabidopsis, all leaves of Col-0 were used (5–6 plants/rep, four reps total). Volatile analysis of freeze-thawing response was carried out similar to wounding, except instead of leaves being cut, they were briefly frozen in liquid N_2_, and immediately placed into the jars for volatile collection. Volatiles were collected for 1 h as previously described.

### Gas Chromatography-Mass Spectrometry

An Agilent 7890B gas chromatograph connected to an Agilent 5977B quadrupole mass spectrometer (Agilent, Santa Clara, CA, United States) was utilized to quantify volatiles. Two μL of liquid sample was injected splitless into a HP-5ms Ultra Inert column (Agilent, Santa Clara, CA, United States). The inlet temperature was set to 240°C for the duration of the run. The oven temperature was as follows: 40°C hold – 2 min, 3°C/min ramp to 160°C, 15°C/min ramp to 280°C, 280°C/min hold – 2 min. The solvent delay was 2.5 min. Analytes were fragmented by positive EI (230°C – source, 150°C – quadrupole, ionization energy – 70 eV, scan range – 25–500 amu). Most compounds were identified and quantified based off of retention times and spectra of pure external standards purchased from Sigma-Aldrich (St. Louis, MO, United States). The 4-oxo-(2*E*)-hexenal, (2*Z*)-pentenyl acetate, pentyl acetate, pentanoic acid, and 2-methyl-3-pentanone were identified based off matching of mass spectra and retention index (RI), calculated according to van Den Dool and Kratz (1963), in the NIST14 library. (2*Z*)-Pentenal and (2*Z*)-hexenal were identified by almost identical spectral matching to (2*E*)-pentenal and (2*E*)-hexenal, respectively, and retention times characteristic of other lipoxygenase-derived volatile (*E*/*Z*)-isomers. All volatiles were quantified based by utilizing internal and external standards.

### Infection Assays

For disease assays, B73 and *lox10-3* mutant maize seedlings at the V4 developmental stage were exposed to PLVs for 2 h before being inoculated 1 h after volatile exposure ended. Six plants of each genotype/treatment were placed into a 6 L glass container along with a cotton ball containing 100 μL of PLV or control treatment. PLV treatment consisted of a mixture containing 10 nmol of (2*Z*)-pentenol and 1-penten-3-ol dissolved in triacetin. Triacetin was used as control treatment. Chemical standards were purchased from Sigma-Aldrich (St. Louis, MO, United States). Plants were inoculated with *C. graminicola* by placing them in humidity chambers and applying 10 μL of spore suspension (10^6^ spores mL^–1^) at six different places on the third leaf of each plant. Plants were removed from humidity chambers 1 day after inoculation and placed back on growth shelves (Gao et al., 2007). For lesion area determination, plants were incubated in conditions as described above for 7 days following inoculation before the infected leaves were excised and scanned to produce digital images. Lesion area were determined from digital images using ImageJ software (Schneider et al., 2012).

For metabolite analysis of infected leaves, B73 plants were either exposed to PLV treatment or control treatment, and infected as described above. Infected leaf tissue was harvested 1 day post inoculation (dpi), 4 dpi, and 6 dpi and stored in –80°C until metabolites were extracted for analysis. Plants exposed to volatile treatments, but were not yet infected, were also collected (0 dpi).

### Metabolite Extraction and Liquid Chromatography-Mass Spectrometry

A mortar and pestle were used to grind frozen plant material into a fine powder under liquid nitrogen. Hormones were extracted from tissue and quantified by liquid chromatography-mass spectrometry (LC-MS/MS) as previously described (Gorman et al., 2020). Five hundred μL of phytohormone extraction buffer (1-propanol/water/HCl [2:1:0.002 vol/vol/vol]) containing 500 nM of the internal standards d-JA (2,4,4-d_3_; acetyl-2,2-d_2_ JA (CDN Isotopes, Pointe-Claire, QC, Canada) and d_6_-SA (Sigma-Aldrich, St. Louis, MO, United States). The samples were agitated in the dark for 30 min at 4°C. Five hundred μL dichloromethane was added to each sample and the samples were again agitated in the dark for 30 min at 4°C. The samples were then centrifuged at 17,000 × *g* for 5 min. The lower organic layer of each sample was transferred to a glass vial and evaporated by nitrogen gas. Samples were resuspended in 150 μL methanol, transferred to a separate tube, and centrifuged at 17,000 × *g* for 5 min to pellet any debris. Ninety μL of supernatant was transferred into autosampler vials for LC-MS/MS.

An Ascentis Express C-18 Column (3 cm × 2.1 mm, 2.7 μm) (Sigma-Aldrich, St. Louis, MO, United States) connected to an API 3200 LC-MS/MS (Sciex, Framingham, MA, United States) using electrospray ionization with multiple reaction mentoring was used. The injection volume was 10 μL and had a 450 μL min^–1^ mobile phase consisting of Solution A (0.2% acetic acid in water) and Solution B (0.2% acetic acid in acetonitrile) with a gradient consisting of (time – %B): 0.5–10%, 1.0–20%, 21.0–70%, 24.6–100%, 24.8–10%, 29 – stop. All oxylipins were quantified by comparison to deuterated internal standards.

## Results

### Spontaneous Catabolism by PLVs

Previously, we analyzed volatiles emitted in B73 inbred WT maize in response to wounding and found a number of PLVs were emitted, although the biosynthetic order of their synthesis was not known (He et al., 2020). To resolve this gap in knowledge and generate a framework of PLV biosynthetic pathway, we incubated plant leaves with various individual PLVs and analyzing subsequent PLV emissions via gas chromatography-mass spectrometry. However, as PLVs are subject to atmospheric breakdown (Orlando et al., 2001), we first attempted to establish which PLVs can be non-enzymatically generated in the absence of plants. To accomplish this, we individually incubated various exogenous PLVs in empty jars, including (2*Z*)-pentenol, (2*E*)-pentenol, (2*E*)-pentenal, pentanol, pentanal, 1-penten-3-ol, 1-penten-3-one, 3-pentanol, or 3-pentanone, and found that several derivative PLVs were subsequently detected. Both (2*Z*)-pentenal and (2*E*)-pentenal were produced after (2*Z*)-pentenol treatment, as well as pentanol (Table 1). Furthermore, these were all produced in equal amounts. In response to (2*E*)-pentenol treatment, (2*E*)-pentenal and pentanol were both produced. Both (2*E*)-butenyl formate and (2*Z*)-butenyl formate were produced in response to (2*E*)-pentenal. Pentanal spontaneously converted to pentanol, as well as to pentanoic acid and to butyl formate. Oppositely, pentanal was produced after pentanol treatment. After 1-penten-3-ol treatment, 1-penten-3-one and 3-pentanol were detected. 3-Pentanone was detected after treatment with 1-penten-3-one, but not after 1-penten-3-ol treatment, despite that 1-penten-3-ol treatment produced 1-penten-3-one. Due to the trace quantities detected after 1-penten-3-one treatment, we concluded that non-enzymatic generation of 3-pentanone from 1-penten-3-one is not of significance. Contrastingly, relatively high amounts of 3-pentanone were detected after 3-pentanol treatment. 3-Pentanol also resulted in the generation of 2-methyl-3-pentanone. These results show that several PLVs can be non-enzymatically generated, although the degree that this process plays in generation of different PLV molecular species varies. Several non-enzymatic PLV derivatives detected in this analysis were not previously reported in either maize (He et al., 2020), Arabidopsis (Mochizuki et al., 2016), or tomato (Shen et al., 2014), including (2*E*)-butenyl formate, (2*Z*)-butenyl formate, butyl formate, pentanoic acid, and 2-methyl-3-pentanone. This suggests that these PLVs are an artifact of the high amounts of exogenous PLVs used in this experiment, and thus are excluded from further consideration in the following plant-based metabolism assays. Other PLVs detected in this experiment are able to be non-enzymatically synthesized. However, these other PLVs have been previously found to be emitted by plant tissues (Shen et al., 2014; Mochizuki et al., 2016; He et al., 2020). We next sought to evaluate how maize leaves metabolize exogenous PLVs.

**TABLE 1:**
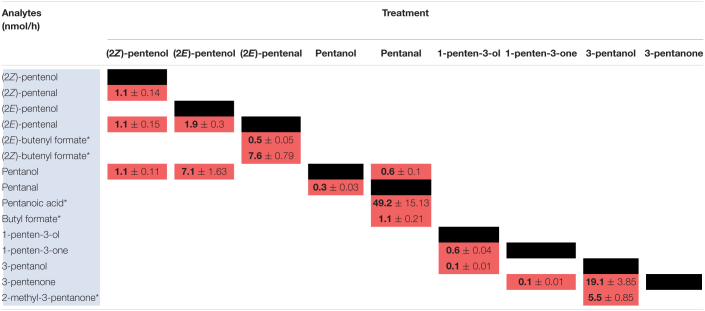
Spontaneous catabolism of exogenous PLVs.

*Various exogenous PLVs were incubated for 20 min in small glass jars before volatiles were collected and analyzed. Analytes detected (red cells) show mean ± SE (nmol per hour). Responses of the same analytes matching a respective volatile treatment were omitted (black cells). Empty cells indicate targets that were not detected. Asterisks after analyte names denote artificial PLVs.*

### Metabolism of PLVs by Maize

**FIGURE 2:**
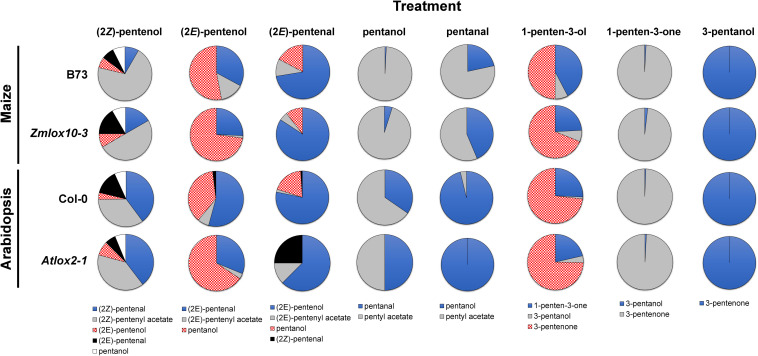
Relative proportions of PLVs detected in maize (B73 and Zm*lox10-3* mutants) and Arabidopsis (Col-0 and *Atlox2-1* mutants) in response to treatment with different PLV molecular species. Colors and patterns representative of different PLVs are shown below individual treatments (top) and are consistent across all genotypes of a particular treatment.

To elucidate maize metabolism of PLVs, we repeated the previous experiment, but we incubated PLVs in the presence of leaves of B73 WT. (2*Z*)-Pentenol was converted into the greatest number of compounds (Table 2 and Figure 2), likely due to its status as a primary PLV. (2*Z*)-Pentenol was most readily converted to (2*Z*)-pentenyl acetate, followed by (2*Z*)-pentenal, (2*E*)-pentenol, (2*E*)-pentenal, and pentanol, which were emitted in approximately equal amounts. (2*E*)-Pentenol exposure also resulted in the emission of (2*E*)-pentenal and pentanol, but produced higher amounts compared to (2*Z*)-pentenol (Table 2). The β-scission reaction proposed by Salch et al. (1995) suggests that pentanol is synthesized from 13*S*-HPOD, but these results show it can also be synthesized by the reduction of 13*S*-HPOT-derived 2-pentenol. Treatment with (2*Z*)-pentenol, (2*E*)-pentenol, and pentanol resulted in the emissions of (2*Z*)-pentenyl acetate, (2*E*)-pentenyl acetate, and pentyl acetate, respectively, suggesting these alcohols can all be acted upon by ATs. Interestingly, (2*Z*)-pentenyl acetate and pentyl acetate were more highly emitted after treatment with (2*Z*)-pentenol and pentanol, respectively, compared to (2*E*)-pentenyl acetate after (2*E*)-pentenol treatment. This suggests that PLV-metabolizing ATs prefer (2*Z*)-pentenol and pentanol as substrates. Importantly, PLV acetate conjugates were not detected after incubation of exogenous PLVs in empty jars, suggesting these PLVs are solely synthesized enzymatically. In addition to pentyl acetate, pentanol treatment also resulted in the emission of the aldehyde, pentanal. Conversely, exposure to pentanal resulted in the emission of pentanol and pentyl acetate. Similarly, (2*E*)-pentenal treatment resulted in the emission of (2*E*)-pentenol, (2*E*)-pentenyl acetate. These results suggest potential bi-directional interconversion of PLV aldehydes and alcohols, as well as the formation of PLV-derived esters. Treatment of B73 with the other primary PLV, 1-penten-3-ol, resulted in the emission of 1-penten-3-one, 3-pentanone, and to a much lesser extent, 3-pentanol. 1-Penten-3-one treatment also resulted in the emission of a small amount of 3-pentanol, and a large amount of 3-pentanone relative to treatment with 1-penten-3-ol. Exposure to 3-pentanol resulted in the emission of 3-pentanone. Oppositely, 3-pentanone resulted in minor emissions of 3-pentanol. No acetate or esterified derivatives of 1-penten-3-ol or any of its derivatives were detected, suggesting that the relevant AT(s) require a terminal hydroxyl group.

**TABLE 2:**
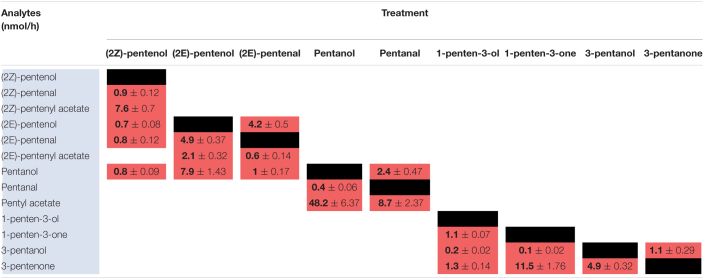
Metabolism of exogenous PLVs by WT maize in the B73 background.

*Leaves of B73 were incubated with various PLVs for 20 min before volatiles were collected and analyzed. Analytes detected (red cells) show mean ± SE (nmol per hour). Responses of the same analytes matching a respective volatile treatment were omitted (black cells). Empty cells indicate targets that were not detected.*

Since PLVs are released from leaves, we also treated PLV-deficient *lox10-3* mutants (He et al., 2020) with PLVs to rule out the emission of endogenous PLVs induced by exogenous PV treatments. Emission of select PLVs in response to treatment with specific PLVs was the same between WT B73 and *lox10-3* mutants (Tables 2, 3), however, some PLVs were more lowly emitted in response to select PLV treatments. Most notably, acetate conjugates were emitted in lower amounts in *lox10-3* mutants relative to B73 WT. This suggests that ZmLOX10 positively regulates AT activity in maize. These results utilizing PLV-deficient *lox10-3* mutants confirm the previous B73 results, and show that ZmLOX10 positively regulates the AT(s) involved in the synthesis of various PLV acetates.

**TABLE 3:**
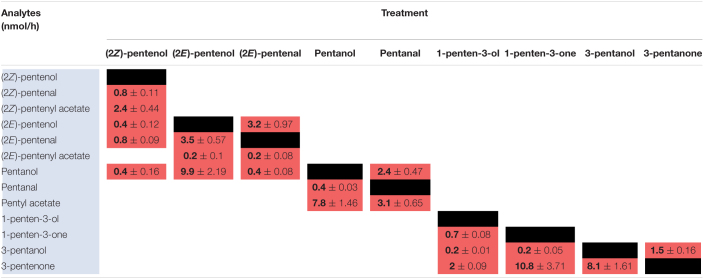
Metabolism of exogenous PLVs by *lox10-3* mutants in the maize B73 background.

*Leaves of lox10-3 mutants were incubated with various PLVs for 20 min before volatiles were collected and analyzed. Analytes detected (red cells) show mean ± SE (nmol per hour). Responses of the same analytes matching a respective volatile treatment were omitted (black cells). Empty cells indicate targets that were not detected.*

### Metabolism of PLVs by Arabidopsis

Green leaf volatiles metabolism can greatly vary between different plant species (Matsui et al., 2000; Allmann and Baldwin, 2010; López-Gresa et al., 2018), therefore we also wanted to evaluate PLV metabolism in Arabidopsis. PLV metabolism was assessed in Arabidopsis Col-0 ecotype as described for maize. We found that overall, the same types of PLVs were emitted in response to select PLV treatments. There were, however, some notable differences regarding the relative amounts of emitted PLVs (Figure 2). After incubation with (2*Z*)-pentenol, equal amounts of (2*Z*)-pentenal and (2*Z*)-pentenyl acetate were recovered, followed by lower amounts of (2*E*)-pentenol and (2*E*)-pentenal (Table 4 and Figure 2). Notably, after (2*E*)-pentenal treatment, large amounts of (2*E*)-pentenol were retrieved. The ratio of pentyl acetate to pentanal produced after pentanol treatment was almost equal (Table 4), as opposed to the large difference observed in B73 maize (Table 2). 1-Penten-3-ol treatment resulted in 2–3 fold higher amounts of 3-pentanone relative to 1-penten-3-one (Table 4). This was substantially different than in maize, which emitted approximately amounts of these volatiles (Tables 2, 3).

**TABLE 4:**
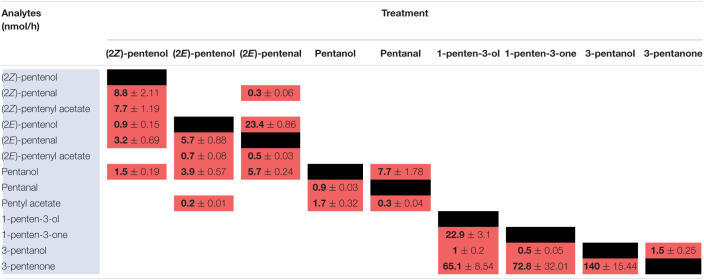
Metabolism of exogenous PLVs by Arabidopsis.

*Intact Arabidopsis, Col-0, were incubated with various PLVs for 20 min before volatiles were collected and analyzed. Analytes detected (red cells) show mean ± SE (nmol per hour). Responses of the same analytes matching a respective volatile treatment were omitted (black cells). Empty cells indicate targets that were not detected.*

As for maize, we also utilized a PLV-deficient mutant of Arabidopsis, *Atlox2-1*, to rule out potential exogenous PLV-mediated induction of endogenous PLV synthesis (Mochizuki et al., 2016). As was the case between B73 WT and *lox10-3* mutant maize, we found few differences in the responses of Col-0 and *Atlox2-1* mutants (Figure 2). After treatment with (2*Z*)-pentenol, less (2*E*)-pentenal was recovered relative to the other metabolites detected than in Col-0 (Tables 4, 5 and Figure 2). After (2*E*)-pentenal treatment, there was less (2*E*)-pentanol and pentanol relative to (2*Z*)-pentenal, and (2*E*)-pentenyl acetate was not detected (Table 5). After pentanol treatment, there were approximately equal amounts of pentyl acetate and pentanal, which differed from the approximate 2:1 ratio of these volatiles found in Col-0. Collectively, these experiments show that PLV metabolism is largely similar in maize and Arabidopsis with regard to the types of individual molecular species that are synthesized in response to individual PLV treatments. However, these experiments also highlight particular PLVs are synthesized at different rates between species (Figure 2), indicating differential enzymatic activity across these two species. Collectively, these experiments establish the biosynthetic order of PLV synthesis within the LOX pathway and provide evidence of enzymatic and non-enzymatic synthesis of PLVs. Despite this, little is known about the enzymes behind PLV synthesis.

**TABLE 5:**
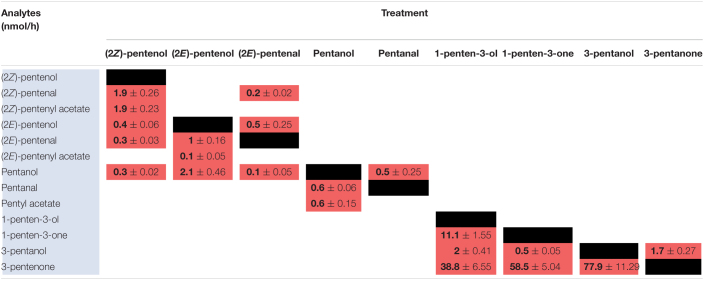
Metabolism of exogenous PLVs by Arabidopsis.

*Intact Arabidopsis, Atlox2-1 mutants in the Col-0 background, were incubated with various PLVs for 20 min before volatiles were collected and analyzed. Analytes detected (red cells) show mean ± SE (nmol per hour). Responses of the same analytes matching a respective volatile treatment were omitted (black cells). Empty cells indicate targets that were not detected.*

### Volatile Analysis of Leaves Reveals Involvement of Different Enzymes in PLV and GLV Metabolism

**FIGURE 3:**
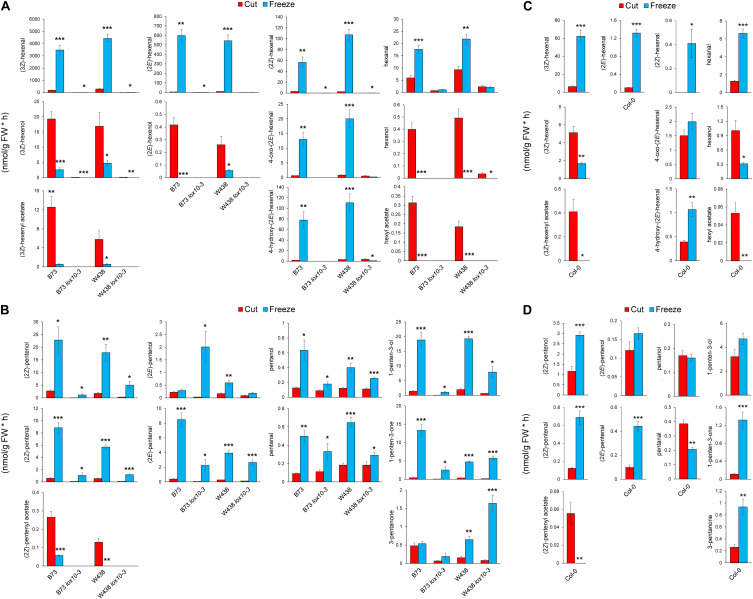
Freezing differentially distorts GLV and PLV emissions of maize and Arabidopsis. Volatiles were collected for 1 h after leaves of two inbred lines of maize, B73 and W438, and *lox10-3* mutants in each respective background, and the Col-0 background of Arabidopsis, were either cut into 1 cm pieces (red) or briefly flash frozen in liquid nitrogen (blue). **(A,C)** Show GLV and **(B,D)** show PLV emissions in maize **(A,B)** and Arabidopsis **(C,D)** (mean ± SE, nmol per gram of fresh weight per hour). Student’s *t*-test was performed to determine statistical difference between treatments for each individual genotype (*p < 0.05, **p < 0.005, ***p < 0.0005) (*n* = 4).

One popular method of volatile elicitation, freeze-thawing, has been reported to irreversibly inactivate GLV-metabolizing ADH, and possibly AT enzymes, which generate GLV alcohol and acetate conjugates, respectively (Fall et al., 2001). However, wounding of plant leaves does not inhibit the function of these enzymes (He et al., 2020). Both GLVs (Bate et al., 1998; Speirs et al., 1998) and PLVs (Gardner et al., 1996) are reported to be acted upon ADHs. GLV aldehydes are also reported to be acted upon by a NADPH-dependent enzyme, CHR (Tanaka et al., 2018). As such, we chose to employ both wounding and freeze-thawing with maize and Arabidopsis in order to determine if synthesis of PLV and GLV alcohols and acetates are similarly affected by freeze-thawing. We started by analyzing GLVs and PLVs from wounded and freeze-thawed leaves of WT and *lox10-3* mutants in the B73 and W438 maize inbred genetic backgrounds. Wounding resulted in the emission of diverse PLVs and GLVs in WTs of both backgrounds, with low amounts emitted by *lox10-3* mutants in both backgrounds (Figure 3A). Notably (2*E*)-pentenyl acetate and pentyl acetate were not emitted in these experiments, indicating that if they are emitted, it is only in trace quantities. Freeze-thawing of leaves resulted in dramatic increases of GLV aldehydes, including (3*Z*)-hexenal, hexanal, (2*E*)-hexenal, 4-hydroxy-(2*E*)-hexenal, 4-oxo-(2*E*)-hexenal (Matsui et al., 2012), and a newly identified GLV, (2*Z*)-hexenal (Figure 3A). Despite the significant increase of aldehyde substrate, freeze-thawing resulted in significantly lower amounts of GLV alcohols, including (3*Z*)-hexenol, (2*E*)-hexenol, and hexanol (Figure 3A). Emissions of the GLV acetates, (3*Z*)-hexenyl acetate and hexyl acetate, were similarly diminished after freeze-thawing.

Oppositely freeze-thawing of leaves resulted in much higher emissions of PLV alcohols, including (2*Z*)-pentenol, 1-penten-3-ol, and pentanol (Figure 3B). An exception to this trend was (2*E*)-pentenol, which was only slightly higher in *lox10-3* mutants in the B73 background, and WT in the W438 background. The PLV aldehydes, (2*Z*)-pentenal, (2*E*)-pentenal, pentanal, as well as the ketone, 1-penten-3-one, were all significantly higher after freeze-thawing in all genotypes. 3-Pentanone was elevated in response to freeze-thawing in WT and *lox10-3* mutants in the W438 background, but not the B73 background (Figure 3B). This represents an interesting difference in PLV synthesis between these different maize inbreds. It is possible that selective pressures have driven this difference, suggesting these volatiles are of consequence to maize fitness in response to different environmental challenges. Like GLV aldehydes, PLVs aldehydes were also more emitted by freeze-thawed leaves, but not to the degree of GLV aldehydes (Figure 3). The sole PLV that was decreased in response freeze-thawing was (2*Z*)-pentenyl acetate (Figure 3B). In fact, (2*Z*)-pentenyl acetate emissions in all genotypes were either completely, or almost completely, abolished by freeze-thawing, suggesting this treatment results in the inactivation of ATs.

**FIGURE 4:**
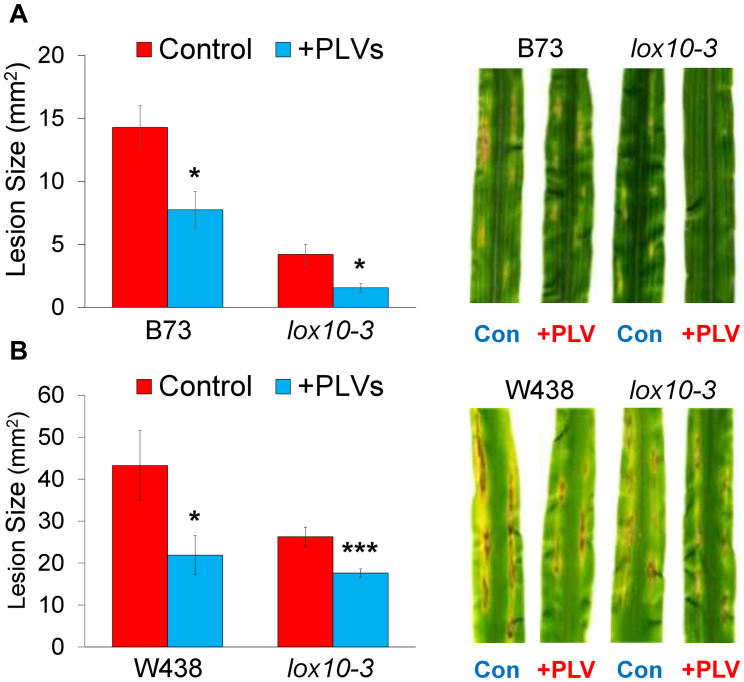
Exogenous treatment with PLVs induces resistance of maize to *C. graminicola.* graph shows the mean area of lesions after inoculation of WT and *lox10-3* mutants in the B73 **(A)** and W438 **(B)** backgrounds after treatment with a PLV mixture containing (2*Z*)-pentenol and 1-penten-3-ol dissolved in triacetin, or control treatment (triacetin). Leaves were harvested after 7 dpi. Student’s *t*-test was performed to determine statistical significance of lesion areas between control and PLV-treated plants within each genotype (*p < 0.005, ***p < 0.0001) (*n* = 6).

To determine if the impact of freeze-thawing on PLV and GLV emissions is consistent throughout diverse plant species, we also analyzed GLVs and PLVs emitted by Arabidopsis Col-0 in response to wounding and freeze-thawing. As seen in maize, wounding elicited the emission of diverse GLVs and PLVs in Arabidopsis (Figures 3C,D). By comparing the relative proportions of maize and Arabidopsis volatiles emitted in response to wounding, it is clear that Arabidopsis favors formation of 1-penten-3-ol and its derivatives, whereas maize favors formation of (2*Z*)-pentenol and its derivatives (Supplementary Figure 1). This suggests that AtLOX2 and ZmLOX6 and/or ZmLOX10 may be able to preferentially direct synthesis of 1-penten-3-ol or (2*Z*)-pentenol from 13*S*-HPOT. GLV and PLV emissions after freeze-thawing, relative to wounding, were similar between maize and Arabidopsis. Aldehyde GLVs were increased, and GLV alcohols and acetates were diminished (Figure 3C). Correspondingly, (2*Z*)-pentenyl acetate was decreased, despite that most other PLVs displayed increased emissions after freeze-thawing (Figure 3D). Only pentanol, pentanal, (2*E*)-pentenol, and 1-penten-3-ol were not statistically increased by freeze-thawing, although the latter two displayed strong trends (Figure 4B). (2*Z*)-Pentenyl acetate was very lowly emitted by both maize and Arabidopsis after freeze-thawing, despite that its precursor, (2*Z*)-pentenol, was highly emitted. Similar responses of PLV and GLV acetates to freeze-thawing, coupled with the structural similarity of these VOCs, increases the likelihood that GLV-producing BAHD ATs also act on PLVs. Inactivation of GLV aldehyde-to-alcohol conversion, but not PLV alcohol-to-aldehyde conversion, suggest that the ADH(s), CHR, or other enzymes acting in these two pathways are different. Collectively, these results shed new light on PLV synthesis in both maize and Arabidopsis. We next focused on the impact of PLV-mediated signaling on maize defense.

### PLVs Induce Maize Resistance to *C. graminicola*

Recently, we had shown that GLVs act as potent promoters of susceptibility to the causal agent of anthracnose disease in maize, *C. graminicola*, a fungal pathogen with a hemi-biotrophic life style (Gorman et al., 2020). Because PLVs are often co-emitted with GLVs (Fall et al., 2001; Heiden et al., 2003; Jardine et al., 2012; He et al., 2020), we examined the role of these volatiles in maize-*C. graminicola* interactions. To determine the impact of PLVs on *C. graminicola* infection, we pre-treated WT and PLV-deficient *lox10-3* mutants with a PLV mixture containing the primary PLVs, (2*Z*)-pentenol and 1-penten-3-ol, before inoculating their leaves. Plants were exposed to these volatiles for 2 h, and inoculated with spores of *C. graminicola* 1 h after volatile exposure ended. Surprisingly, we found that PLV treatment significantly increased resistance in WT B73 and W438 (Figure 4), the opposite effect observed after GLV treatment (Gorman et al., 2020). Surprisingly, *lox10-3* mutants in both backgrounds, which already display resistance to *C. graminicola* (Gorman et al., 2020; Wang et al., 2020a), had their resistance further enhanced by PLV exposure (Figure 4). Previously, we had shown that *lox10-3* mutant resistance is due to low levels of the biologically active jasmonate, jasmonic acid-isoleucine (JA-Ile), and elevated concentrations of salicylic acid (SA), the phytohormone known to govern resistance against (hemi)-biotrophic pathogens. Furthermore, we found that exogenous application of SA was unable to induce further resistance in these mutants (Gorman et al., 2020). This suggests that SA-mediated resistance is already saturated in these mutants. Taking this into account, it is likely that PLV-mediated induction of resistance is not related to increases or decreases of SA or JA-Ile, respectively.

### PLV-Mediated Resistance to *C. graminicola* Correlates With Increased Synthesis of Oxylipin Hydroxides and α- and γ-Ketols

**FIGURE 5:**
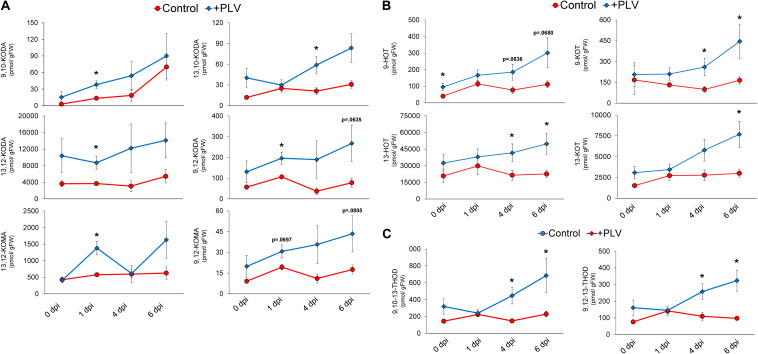
Pentyl leaf volatiles induce synthesis of oxylipin ketols, hydroxytrienes, ketotrienes, and trihydroxydienes in response to *C. graminicola* infection. The graph shows the mean amount of various oxylipin ketols **(A)** and hydroxytrienes and ketotrienes **(B)** and trihydroxydienes **(C)** throughout *C. graminicola* infection of B73 leaves after treatment with a PLV mixture [(2*Z*)-pentenol and 1-penten-3-ol] dissolved in triacetin, or control treatment (triacetin). Plants were exposed to treatments for 2 h prior to inoculation and inoculated with *C. graminicola* spores 1 h after volatile exposure was halted. Oxylipins were analyzed after 1, 4, and 6 days post inoculation (dpi), as well as before infection, 0 dpi. Student’s *t*-test was performed to determine statistical significance between control and PLV-treated plants at each timepoint (*p* < 0.05) (*n* = 6).

In order to further investigate the potential biochemical mechanisms underlying PLV-mediated resistance to *C. graminicola* in maize, we used liquid chromatography mass spectrometry to measure a variety of different phytohormones and oxylipin metabolites in maize leaves infected with *C. graminicola* after treatment with PLVs. As hypothesized, the levels of JA-Ile and SA were not different between control and PLV-treated plants (Supplementary Figure 2). However, several 9- and 13-LOX-derived α- and γ-ketols, produced in the allene oxide synthase (AOS) branch of the LOX pathway, were higher in the infected plants exposed to PLVs (Figure 5A). Most ketols appeared to be elevated in the PLV-treated plants throughout the duration of infection, but many of these ketols, including 9-hydroxy-10-oxo-(12*Z*,15*Z*)-octadecadienoic acid (9,10-KODA), 9-hydroxy-12-oxo-(10*E*,15*Z*)-octadecadienoic acid (9,12-KODA), 13-hydroxy-12-oxo-(9*Z*,15*Z*)-octadecadienoic acid (13,12-KODA), and 13-hydroxy-12-oxo-(9*Z*)-octadecadienoic acid (13,12-KOMA), were most significantly increased at 1 day post inoculation (dpi). 9-hydroxy-12-oxo-(10*E*)-octadecenoic acid (9,12-KOMA) appeared to be higher at most timepoints in infected PLV-treated plants, but was not statistically higher, though it was close at 1 dpi (*p* = 0.0697) and 6 dpi (*p* = 0.0805). Only 13-hydroxy-10-oxo-(11*E*,15*Z*)-octadecadienoic acid (13,10-KODA) did not come close to statistical difference at this timepoint, but was significantly higher in PLV-treated plants at 4 dpi. Importantly, we have previously shown that exogenous treatments with several of these ketols, including the α-ketol 9,10-KODA (Wang et al., 2020a), and the γ-ketols, 9,12-KOMA and 9,12-KODA (Wang et al., 2020b), strongly increase maize resistance to *C. graminicola*.

In addition to LOX-derived oxylipin ketols, several 13*S*-HPOT-derived oxylipin hydroxytrienes and ketotrienes were increased in PLV-treated plants throughout the course of infection (Figure 5B). Other oxylipins were also increased over the course of infection in plants exposed to PLVs. This includes 9-hydroxy-(10*E*,12*Z*,15*Z*)-octadecatrienoic acid (9-HOT) and 13-hydroxy-(9*Z*,11*E*,15*Z*)-octadecatrienoic acid (13-HOT), produced in the reductase (RED) and peroxygenase (POX) branches of the LOX pathway (Blée, 2002), and 9-oxo-(10*E*,12*Z*,15*Z*)-octadecatrienoic acid (9-KOT) and 13-oxo-(9*Z*,11*E*,15*Z*)-octadecatrienoic acid (13-KOT), which are produced in the LOX (Vollenweider et al., 2000), RED, and POX (Chechetkin et al., 2004) branches of the LOX pathway. Collectively, the greatest differences in these oxylipins between control and PLV-treated plants were observed at the two latest timepoints, 4 and 6 dpi, though there were some exceptions. 9-HOT was already induced by PLV treatment prior to infection, 0 dpi, but was not statistically higher at later timepoints, though it was close at 4 dpi (*p* = 0.0636) and 6 dpi (*p* = 0.0680). Additionally, while 13-KOT was statistically higher in PLV-treated plants at 6 dpi, it was not at 4 dpi. Lastly, 9,10,13-trihydroxy-(11*E*,15*Z*)-octadecadienoic acid (9,10,13-THOD) and 9,12,13-trihydroxy-(10*E*,15*Z*)-octadecadienoic acid (9,12,13-THOD), two trihydroxydienes produced in the epoxy alcohol synthase (EAS) branch of the LOX pathway (Blée, 2002), were also higher at 4 and 6 dpi in PLV-treated plants (Figure 5C). Collectively, these results suggest that several oxylipins, including α- and γ-ketols, ketotrienes, hydroxytrienes, and trihydroxydienes, are involved in PLV-mediated defense against *C. graminicola* in maize.

## Discussion

Pentyl leaf volatiles represent a widely emitted group of plant volatiles, though little is known regarding their synthesis or biological roles. Aspects of PLV metabolism had been previously hypothesized (Gardner et al., 1996), but lacked a full experimental examination of the pathway. In order to determine the biosynthetic order of individual PLVs, we exposed both maize and Arabidopsis to exogenous PLVs and analyzed subsequent PLV emissions. Deuterated exogenous PLV standard are not available, therefore, in order to demostrate that PLVs detected in these experiments were from exogenous PLV standards and not endogenous PLVs, we applied high concentrations of exogenous PLVs and employed the use of PLV-deficient mutants in both maize, *lox10-3* (He et al., 2020) and Arabidopsis, *Atlox2-1* (Mochizuki et al., 2016). Treatment with (2*Z*)-pentenol and 1-penten-3-ol produced most other PLVs (Tables 2–5), validating their predicted status as primary PLVs (Salch et al., 1995). Importantly, no exogenous PLV treatments resulted in the production of either of these volatiles, suggesting that PLV-mediated signaling does not induce PLV biosynthesis. This observation, in addition to the utilization of PLV-deficient mutants, confirmed that PLVs detected after treatment are derived from exogenous PLVs. The artificially high amount of PLVs used in these experiments also made it necessary to determine which PLVs detected in these experiments were a result of plant absorption, metabolism, and re-emission, and which were produced by non-enzymatic degradation unrelated to plant tissues. By incubating PLVs in the absence of plants, we identified several artificial PLVs (Table 1), including pentanoic acid, but(en)yl formates, 3-methyl-2-pentanone, and excluded them from further analyses. We also identified several PLVs that were non-enzymatically synthesized, but have been previously reported to be emitted by plant tissues (Shen et al., 2014; Mochizuki et al., 2016; He et al., 2020). Though these PLVs are able to be non-enzymatically generated, the impact of enzymatic vs. non-enzymatic synthesis of these PLVs *in planta* is difficult to discern, as PLVs are known to be sequestered in plant tissues by binding to non-volatile metabolites (Sugimoto et al., 2015).

**FIGURE 6:**
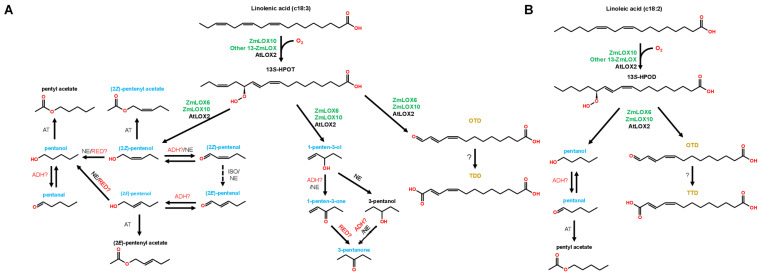
Working model of the PLV pathway in maize (green gene names) and Arabidopsis (black gene names). The graph shows both C18:3-derived **(A)** and C18:2-derived **(B)** PLV synthesis. Dashed arrows indicate hypothetical reactions. Alcohol dehydrogenase (ADH), acetyl transferase (AT), reductase (RED), non-enzymatic (NE), isomerase (ISO). The names of biologically relevant PLVs are blue, the names of trace PLVs are black, and the names of 13-carbon compounds (Gao et al., 2008) are orange. Adapted from Gardner et al. (1996).

Treatment with all PLV alcohols, including (2*Z*)-pentenol, (2*E*)-pentenol, 1-penten-3-ol, and pentanol, resulted in their oxidation to their respective aldehyde and ketone derivatives (Tables 2–5). Previously, generation of (2*Z*)-pentenal and (2*E*)-pentenal from (2*Z*)-pentenol was reported to be catalyzed by an ADH(s) (Gardner et al., 1996), however, we also found that non-enzymatic generation plays a part in their generation (Table 1). This was also true of pentanal and 3-pentanone generated from pentanol and 3-pentanol, respectively. Prior analysis of atmospheric decomposition by PLV alcohols supports these findings (Orlando et al., 2001). Interestingly, while conversion of (2*E*)-pentenol to (2*E*)-pentenal occurred, the opposite reaction also occurred, suggesting that stoichiometry dictates the observed reaction (Tables 2–5). This was also true of pentanol and pentanal. Interestingly, various alkene PLVs were reduced to alkanes. This was unexpected, as reduction of carbon-carbon double bonds in GLVs, which are better characterized and similarly structured (Figure 1), has not been reported. These reactions seemed to be largely driven by spontaneous, non-enzymatic decomposition of PLVs (Table 1), though this does not rule out the possible existence of a PLV-specific reductase. Similar to GLVs, PLV alcohols are able to be metabolized into more chemically stable acetate conjugates (Tables 2–5), likely through the activity of the same AT(s). Notably, the relevant ATs are more active on PLV alkenes than alkanes, and requires PLV substrate to possess a terminal hydroxyl group. In response to 1-penten-3-ol and 3-pentanol, 1-penten-3-one and 3-pentanone appeared more readily synthesized by Arabidopsis (Tables 2–5 and Figure 2). Conversely, maize seemed to produce more PLV acetate conjugates in response to volatile treatments. These observations were supported by observations of volatile emissions between the two species (Figure 3). These observations suggest selective pressure on PLV synthesis in plants for, as of yet, unknown purposes. When comparing the overall effect of ZmLOX10 and AtLOX2 on PLV metabolism in maize and Arabidopsis, respectively, the only notable difference was the relative quantity of PLV acetates, with both mutants possessing lesser proportions of these volatiles in response to treatment with various PLV alcohols (Figure 2). Both of these LOX isoforms are the major 13-oxylipin producers in their respective species, and one or many of these oxylipins could directly or indirectly affect expression or function of AT(s) relevant to GLV and PLV synthesis. Based on these collective results, we have summarized the biosynthetic pathway of PLVs in Figure 6.

GLV and PLV analysis of wounded and freeze-thawed leaves of maize and Arabidopsis helped establish the biosynthetic order of PLVs, as well as identify PLVs that are most likely relevant to biological processes. We found that wounding of both species resulted in emission of several GLVs and PLVs (Figure 3). We were surprised to find GLV emissions in Arabidopsis Col-0, which was reported to possess a natural mutation in its AtHPL1 gene that renders it non-functional (Duan et al., 2005), though the quantities were very low. This analysis also confirmed that ZmLOX10 is critical for GLV and PLV synthesis in both the W438 and B73 backgrounds (Christensen et al., 2013; He et al., 2020). We also identified a GLV, (2*Z*)-hexenal, and a PLV, (2*Z*)-pentenyl acetate, that have not been previously reported to be emitted by plants. Furthermore, these volatiles were emitted by both Arabidopsis and maize. Emissions of (2*Z*)-pentenol and (2*E*)-pentenal in both species strongly correlated, but not with (2*E*)-pentenol. This indicates that (2*E*)-pentenol is likely derived from (2*E*)-pentenal, which is in turn derived from (2*Z*)-pentenol, rather than from (2*Z*)-pentenol directly. Several PLVs identified in the PLV metabolism assays were not found in volatile analysis of plant tissues, including (2*E*)-pentenyl acetate, pentyl acetate, and 3-pentanol (Figure 3). This suggests that they are likely only synthesized and emitted in trace quantities.

Another important finding from this study is that the widely used method of volatile analyses by freeze-thawing tissues may lead to consequential distortions of volatile profiles. These two methods differ in the number of cells they disrupt, with freeze-thawing disturbing all cells in the leaf tissue and wounding disturbing a fewer number of cells. Volatile elicitation by complete tissue disruption, including freeze-thawing, is often used for analysis of volatiles, despite that it has been previously reported to eliminate emissions of GLV alcohols, likely through inactivation of enzymes involved in volatile metabolism, such as ADH (Fall et al., 2001; Matsui et al., 2012) and CHR (Tanaka et al., 2018). ADHs (Bate et al., 1998; Speirs et al., 1998) and CHR (Tanaka et al., 2018), which utilize the cofactors NADH and NADPH, respectively, are known to facilitate conversion of GLV aldehydes to alcohols. Importantly, CHR was found to be more important for metabolism of aldehydes in intact tissues (Tanaka et al., 2018). ADHs are also reported to convert PLV alcohols to aldehydes (Gardner et al., 1996), though very little is known about these enzymes. Thus, we expected emission of PLV aldehydes and GLV alcohols and acetate conjugates to be low in response to freeze-thawing. This hypothesis was only partially supported, with freeze-thawing resulting in significantly diminished levels of GLV alcohols and acetates (Figure 3). Conversely, both GLV and PLV aldehydes, as well as PLV alcohols, were increased in response to freeze-thawing (Figure 3). This suggests that despite ADHs having reported activity in both GLV and PLV metabolism, and the structural similarity of these two groups, these reactions are potentially catalyzed by different enzymes. GLV and PLV alcohols did not share similar responses to freeze-thawing, but emissions of acetate conjugates of both groups were significantly reduced (Figure 3). This suggests that the AT(s) that acts on GLVs may also metabolize PLVs. Additionally, since (2*Z*)-pentenyl acetate is derived from (2*Z*)-pentenol, which was more highly emitted after freeze-thawing, these results suggest that function of both ADHs and ATs are negatively impacted by freezing. The exact identity of the enzymes involved in PLV metabolism remains unknown and should be further investigated. ADHs and BAHD ATs constitute large families of enzymes, making it likely that a wide range of volatiles are impacted by freeze-thawing. Taking this into account, utilization of freeze-thawing to elicit volatile emissions should be used with caution in the future.

Though GLVs and PLVs are often co-emitted, they also share clear antagonism. This is evidenced by increases in PLV emission in *hpl* mutants (Vancanneyt et al., 2001; Salas et al., 2006; Shen et al., 2014). Since GLV and PLV synthesis requires substrate from the same LOXs, this is not surprising. Previously, both GLVs and PLVs have been implicated in susceptibility (Scala et al., 2013) and resistance (Song et al., 2015) of Arabidopsis to infection by *P. syringae*, respectively. In this study, we found that PLVs induced greater resistance to *C. graminicola* in two inbred backgrounds of maize (Figure 4). Furthermore, PLVs were able to further enhance resistance in already-resistant *lox10-3* mutants in both inbred backgrounds. Previously, we revealed that GLVs strongly promoted anthracnose disease progression caused by this same pathogen through induction of JA-Ile and suppression of SA (Gorman et al., 2020). Upon investigation of the biochemical mechanisms underlying PLV-mediated resistance, we did not find that JA-Ile or SA levels were significantly altered in PLV-treated plants at any point throughout infection (Supplementary Figure 2). Despite this, it is still possible these phytohormones are involved in PLV-mediated responses to other stresses. However, we did find that a variety of LOX-derived oxylipins, including α- and γ-ketols, ketotrienes, hydroxytrienes, and trihydroxydienes, were increased in PLV-treated plants throughout the time course of infection (Figure 5). Though there were slight differences, most ketols were significantly increased at the earliest timepoint after inoculation, 1 dpi (Figure 5A). Contrarily, ketotrienes, hydroxytrienes, and trihydroxydienes were, overall, significantly elevated in PLV-treated plants at later timepoints in the infection process, 4 and 6 dpi (Figures 5B,C). We previously showed that the α-ketol 9,10-KODA (Wang et al., 2020a), and the γ-ketols, 9,12-KOMA and 9,12-KODA (Wang et al., 2020b), act as potent priming agents that induce resistance to *C. graminicola*. The ketotriene, 9-KOT, has been shown to provide resistance to pathogens (Vicente et al., 2012) as well as inhibit their *in vitro* vegetative growth (Prost et al., 2005). The hydroxytriene, 9-HOT, is involved in the regulation of defense gene expression (Vellosillo et al., 2007). Importantly, *C. graminicola* switches from a phase of biotrophic growth to necrotrophic growth approximately 48–72 h after inoculation, where it begins to form visible lesions (Bergstrom and Nicholson, 1999; Mims and Vaillancourt, 2002; Vargas et al., 2012). Collectively, these results indicate that ketols may be effective at mediating resistance to this pathogen during the biotrophic phase of infection by *C. graminicola*, and ketotrienes, hydroxytrienes, and trihydroxydienes may be involved in defense during its necrotrophic phase of growth. Determining the specific roles of these oxylipins in maize-*C. graminicola* interactions should be the subject of future studies. These results support previous findings that PLVs induce resistance to biotrophic and hemi-biotrophic pathogens (Song and Ryu, 2013; Choi et al., 2014; Song et al., 2015), and provide another example of their antagonism to GLVs.

Green leaf volatiles emissions are known to significantly vary in composition and abundance across the plant kingdom (Engelberth and Engelberth, 2020), and this is likely also the case for PLV emissions because of their biosynthetic antagonism with GLVs (Vancanneyt et al., 2001; Salas et al., 2006; Shen et al., 2014). These differences in volatile emissions are likely a result of selective pressures that each plant species has faced throughout their respective evolutionary histories. For example, GLVs mediate maize susceptibility to *C. graminicola* and other hemi-biotrophic pathogens (Gorman et al., 2020; Wang et al., 2020a), but GLVs are critical for defense against insect herbivory (Rojas et al., 2018). Maize is one of the highest GLV-producing monocots (Engelberth and Engelberth, 2020) and has historically experienced significant insect herbivory, suggesting that GLV-mediated susceptibility to *C. graminicola* could be an unintended consequence of evolutionary response to insect pressure or breeding efforts. Oppositely, there are some plants that emit little-to-no GLVs (Engelberth and Engelberth, 2020), and are therefore likely to have increased PLV emissions, which could be a consequence of greater biotrophic or hemi-biotrophic pathogen pressures. As of now, these hypotheses remain untested, and should be the focus of future studies.

This work provides a solid framework of the PLV metabolic pathway in plants that future studies can build upon to elucidate the enzymes involved in PLV synthesis, and establish additional biological effects of these volatiles. Importantly, this work suggests that structure of the PLV pathway is conserved across diverse plant species, but that the enzymes involved in PLV metabolism can vary, even across different genetic backgrounds of the same species. This suggests selective pressure on PLV synthesis, which is supported by their ability to induce resistance to pathogens and insects, including the economically costly maize pathogen, *C. graminicola.* Ultimately, this work will facilitate future studies into this widely emitted, yet enigmatic group of oxylipin volatiles.

## Data Availability Statement

The original contributions presented in the study are included in the article/Supplementary Material, further inquiries can be directed to the corresponding author.

## Author Contributions

ZG and MK designed the all experiments. ZG executed the all experiments, performed the all analyses, and wrote the manuscript. JT grew and screened Arabidopsis for the experiments. All the authors reviewed the manuscript.

## Conflict of Interest

The authors declare that the research was conducted in the absence of any commercial or financial relationships that could be construed as a potential conflict of interest.

## Publisher’s Note

All claims expressed in this article are solely those of the authors and do not necessarily represent those of their affiliated organizations, or those of the publisher, the editors and the reviewers. Any product that may be evaluated in this article, or claim that may be made by its manufacturer, is not guaranteed or endorsed by the publisher.

## Footnotes

1
https://www.pioneer.com

## References

[B1] Allmann S. Baldwin I. T. (2010). Insects betray themselves in nature to predators by rapid isomerization of green leaf volatiles. *Science* 329 1075–1078. 10.1126/science.1191634 20798319

[B2] Alméras E. Stolz S. Vollenweider S. Reymond P. Mène-Saffrané L. Farmer E. E. (2003). Reactive electrophile species activate defense gene expression in *Arabidopsis*. *Plant J.* 34 205–216. 12694595 10.1046/j.1365-313x.2003.01718.x

[B3] Ameye M. Allmann S. Verwaeren J. Smagghe G. Haesaert G. Schuurink R. C. (2018). Green leaf volatile production by plants: a meta-analysis. *New Phytol.* 220 666–683. 10.1111/nph.14671 28665020

[B4] Bate N. J. Riley J. C. Thompson J. E. Rothstein S. J. (1998). Quantitative and qualitative differences in C6-volatile production from the lipoxygenase pathway in an alcohol dehydrogenase mutant of *Arabidopsis thaliana*. *Physiol. Plant.* 104 97–104. 10.1034/j.1399-3054.1998.1040113.x 11841302

[B5] Bergstrom G. C. Nicholson R. L. (1999). The biology of corn anthracnose: knowledge to exploit for improved management. *Plant Dis.* 83 596–608. 10.1094/pdis.1999.83.7.596 30845609

[B6] Blée E. (2002). Impact of phyto-oxylipins in plant defense. *Trends Plant Sci.* 7 315–322. 10.1016/s1360-1385(02)02290-2 12119169

[B7] Chechetkin I. R. Medvedeva N. V. Grechkin A. N. (2004). The novel pathway for ketodiene oxylipin biosynthesis in Jerusalem artichoke (*Helianthus tuberosus*) tubers. *Biochim. Biophys. Acta BBA Mol. Cell Biol. Lipids* 1686 7–14. 10.1016/j.bbalip.2004.07.001 15522817

[B8] Choi H. K. Song G. C. Yi H. S. Ryu C. M. (2014). Field evaluation of the bacterial volatile derivative 3-pentanol in priming for induced resistance in pepper. *J. Chem. Ecol.* 40 882–892. 10.1007/s10886-014-0488-z 25149655

[B9] Christensen S. A. Nemchenko A. Borrego E. Murray I. Sobhy I. S. Bosak L. (2013). The maize lipoxygenase, ZmLOX10, mediates green leaf volatile, jasmonate and herbivore-induced plant volatile production for defense against insect attack. *Plant J.* 74 59–73. 10.1111/tpj.12101 23279660

[B10] D’Auria J. C. Pichersky E. Schaub A. Hansel A. Gershenzon J. (2007). Characterization of a BAHD acyltransferase responsible for producing the green leaf volatile (Z)-3-hexen-1-yl acetate in *Arabidopsis thaliana*. *Plant J.* 49 194–207. 10.1111/j.1365-313x.2006.02946.x 17163881

[B11] Duan H. Huang M. Y. Palacio K. Schuler M. A. (2005). Variations in CYP74B2 (hydroperoxide lyase) gene expression differentially affect hexenal signaling in the Columbia and Landsberg erecta ecotypes of *Arabidopsis*. *Plant Physiol.* 139 1529–1544. 10.1104/pp.105.067249 16258015 PMC1283787

[B12] Engelberth J. Alborn H. T. Schmelz E. A. Tumlinson J. H. (2004). Airborne signals prime plants against insect herbivore attack. *Proc. Natl. Acad. Sci. U.S.A.* 101 1781–1785. 10.1073/pnas.0308037100 14749516 PMC341853

[B13] Engelberth J. Engelberth M. (2020). Variability in the capacity to produce damage-induced aldehyde green leaf volatiles among different plant species provides novel insights into biosynthetic diversity. *Plants* 9:213. 10.3390/plants9020213 32041302 PMC7076675

[B14] Fall R. Karl T. Jordan A. Lindinger W. (2001). Biogenic C5 VOCs: release from leaves after freeze–thaw wounding and occurrence in air at a high mountain observatory. *Atmos. Environ.* 35 3905–3916. 10.1016/s1352-2310(01)00141-8

[B15] Fisher A. J. Grimes H. D. Fall R. (2003). The biochemical origin of pentenol emissions from wounded leaves. *Phytochemistry* 62 159–163. 10.1016/s0031-9422(02)00521-6 12482451

[B16] Gao X. Shim W. B. Göbel C. Kunze S. Feussner I. Meeley R. (2007). Disruption of a maize 9-lipoxygenase results in increased resistance to fungal pathogens and reduced levels of contamination with mycotoxin fumonisin. *Mol. Plant Microbe Interact.* 20 922–933. 10.1094/mpmi-20-8-0922 17722696

[B17] Gao X. Stumpe M. Feussner I. Kolomiets M. (2008). A novel plastidial lipoxygenase of maize (Zea mays) ZmLOX6 encodes for a fatty acid hydroperoxide lyase and is uniquely regulated by phytohormones and pathogen infection. *Planta* 227 491–503. 10.1007/s00425-007-0634-8 17922288

[B18] Gardner H. W. Grove M. J. Salch Y. P. (1996). Enzymic pathway to ethyl vinyl ketone and 2-pentenal in soybean preparations. *J. Agric. Food Chem.* 44 882–886. 10.1021/jf950509r

[B19] Glauser G. Dubugnon L. Mousavi S. A. Rudaz S. Wolfender J. L. Farmer E. E. (2009). Velocity estimates for signal propagation leading to systemic jasmonic acid accumulation in wounded *Arabidopsis*. *J. Biol. Chem.* 284 34506–34513. 10.1074/jbc.m109.061432 19846562 PMC2787311

[B20] Gorman Z. Christensen S. A. Yan Y. He Y. Borrego E. Kolomiets M. V. (2020). Green leaf volatiles and jasmonic acid enhance susceptibility to anthracnose diseases caused by *Colletotrichum graminicola* in maize. *Mol. Plant Pathol.* 21 702–715. 10.1111/mpp.12924 32105380 PMC7170777

[B21] Hatanaka A. (1993). The biogeneration of green odour by green leaves. *Phytochemistry* 34 1201–1218. 10.1016/0031-9422(91)80003-j

[B22] He Y. Borrego E. J. Gorman Z. Huang P. C. Kolomiets M. V. (2020). Relative contribution of LOX10, green leaf volatiles and JA to wound-induced local and systemic oxylipin and hormone signature in Zea mays (maize). *Phytochemistry* 174:112334. 10.1016/j.phytochem.2020.112334 32172019

[B23] Heiden A. C. Kobel K. Langebartels C. Schuh-Thomas G. Wildt J. (2003). Emissions of oxygenated volatile organic compounds from plants Part I: emissions from lipoxygenase activity. *J. Atmos. Chem.* 45 143–172.

[B24] Jardine K. Barron-Gafford G. A. Norman J. P. Abrell L. Monson R. K. Meyers K. T. (2012). Green leaf volatiles and oxygenated metabolite emission bursts from mesquite branches following light–dark transitions. *Photosynth. Res.* 113 321–333. 10.1007/s11120-012-9746-5 22711426

[B25] Kobayashi A. Tsuda Y. Hirata N. Kubota K. Kitamura K. (1995). Aroma constituents of soybean [*Glycine max* (L.) Merril] milk lacking lipoxygenase isoenzymes. *J. Agric. Food Chem.* 43 2449–2452. 10.1021/jf00057a025

[B26] Kunishima M. Yamauchi Y. Mizutani M. Kuse M. Takikawa H. Sugimoto Y. (2016). Identification of (Z)-3:(E)-2-hexenal isomerases essential to the production of the leaf aldehyde in plants. *J. Biol. Chem.* 291 14023–14033. 10.1074/jbc.m116.726687 27129773 PMC4933162

[B27] López-Gresa M. P. Payá C. Ozáez M. Rodrigo I. Conejero V. Klee H. (2018). A new role for green leaf volatile esters in tomato stomatal defense against *Pseudomonas syringe* pv. tomato. *Front. Plant Sci.* 9:1855. 10.3389/fpls.2018.01855 30619420 PMC6305539

[B28] Loughrin J. H. Manukian A. Heath R. R. Tumlinson J. H. (1995). Volatiles emitted by different cotton varieties damaged by feeding beet armyworm larvae. *J. Chem. Ecol.* 21 1217–1227. 10.1007/bf02228321 24234527

[B29] Matsui K. (2006). Green leaf volatiles: hydroperoxide lyase pathway of oxylipin metabolism. *Curr. Opin. Plant Biol.* 9 274–280. 10.1016/j.pbi.2006.03.002 16595187

[B30] Matsui K. Sugimoto K. Mano J. I. Ozawa R. Takabayashi J. (2012). Differential metabolisms of green leaf volatiles in injured and intact parts of a wounded leaf meet distinct ecophysiological requirements. *PLoS One* 7:e36433. 10.1371/journal.pone.0036433 22558466 PMC3340338

[B31] Matsui K. Ujita C. Fujimoto S. H. Wilkinson J. Hiatt B. Knauf V. (2000). Fatty acid 9-and 13-hydroperoxide lyases from cucumber. *FEBS lett.* 481, 183–188. 10.1016/s0014-5793(00)01997-9 10996320

[B32] Mims C. W. Vaillancourt L. J. (2002). Ultrastructural characterization of infection and colonization of maize leaves by *Colletotrichum graminicola*, and by a *C. graminicola* pathogenicity mutant. *Phytopathology* 92 803–812. 10.1094/phyto.2002.92.7.803 18943278

[B33] Mochizuki S. Sugimoto K. Koeduka T. Matsui K. (2016). *Arabidopsis* lipoxygenase 2 is essential for formation of green leaf volatiles and five-carbon volatiles. *FEBS Lett.* 590 1017–1027. 10.1002/1873-3468.12133 26991128

[B34] Mukhtarova L. S. Brühlmann F. Hamberg M. Khairutdinov B. I. Grechkin A. N. (2018). Plant hydroperoxide-cleaving enzymes (CYP74 family) function as hemiacetal synthases: structural proof of hemiacetals by NMR spectroscopy. *Biochim. Biophys. Acta BBA Mol. Cell Biol. Lipids* 1863 1316–1322. 10.1016/j.bbalip.2018.08.011 30305246

[B35] Orlando J. J. Tyndall G. S. Ceazan N. (2001). Rate coefficients and product yields from reaction of OH with 1-penten-3-ol, (Z)-2-penten-1-ol, and allyl alcohol (2-propen-1-ol). *J. Phys. Chem. A* 105 3564–3569. 10.1021/jp0041712

[B36] Prost I. Dhondt S. Rothe G. Vicente J. Rodriguez M. J. Kift N. (2005). Evaluation of the antimicrobial activities of plant oxylipins supports their involvement in defense against pathogens. *Plant Physiol.* 139 1902–1913. 10.1104/pp.105.066274 16299186 PMC1310568

[B37] Roberts J. M. Kundun J. Rowley C. Hall D. R. Douglas P. Pope T. W. (2019). Electrophysiological and behavioral responses of adult vine weevil, *Otiorhynchus sulcatus* (Coleoptera: Curculionidae), to host plant odors. *J. Chem. Ecol.* 45 858–868. 10.1007/s10886-019-01108-x 31637564

[B38] Rojas J. C. Kolomiets M. V. Bernal J. S. (2018). Nonsensical choices? Fall armyworm moths choose seemingly best or worst hosts for their larvae, but neonate larvae make their own choices. *PloS One* 13:e0197628. 10.1371/journal.pone.0197628 29795622 PMC5967860

[B39] Salas J. J. García-González D. L. Aparicio R. (2006). Volatile compound biosynthesis by green leaves from an *Arabidopsis thaliana* hydroperoxide lyase knockout mutant. *J. Agric. Food Chem.* 54 8199–8205. 10.1021/jf061493f 17032029

[B40] Salch Y. P. Grove M. J. Takamura H. Gardner H. W. (1995). Characterization of a C-5, 13-cleaving enzyme of 13 (S)-hydroperoxide of linolenic acid by soybean seed. *Plant Physiol.* 108 1211–1218. 10.1104/pp.108.3.1211 12228538 PMC157475

[B41] Scala A. Mirabella R. Mugo C. Matsui K. Haring M. A. Schuurink R. C. (2013). E-2-hexenal promotes susceptibility to Pseudomonas syringae by activating jasmonic acid pathways in *Arabidopsis*. *Front. Plant Sci.* 4:74. 10.3389/fpls.2013.00074 23630530 PMC3624080

[B42] Schneider C. A. Rasband W. S. Eliceiri K. W. (2012). NIH image to imageJ: 25 years of image analysis. *Nat. Methods* 9:671. 10.1038/nmeth.2089 22930834 PMC5554542

[B43] Seo H. S. Song J. T. Cheong J. J. Lee Y. H. Lee Y. W. Hwang I. (2001). Jasmonic acid carboxyl methyltransferase: a key enzyme for jasmonate-regulated plant responses. *Proc. Natl. Acad. Sci. U.S.A.* 98 4788–4793. 10.1073/pnas.081557298 11287667 PMC31912

[B44] Shen J. Tieman D. Jones J. B. Taylor M. G. Schmelz E. Huffaker A. (2014). A 13-lipoxygenase, TomloxC, is essential for synthesis of C5 flavour volatiles in tomato. *J. Exp. Bot.* 65 419–428. 10.1093/jxb/ert382 24453226 PMC3904703

[B45] Song G. C. Choi H. K. Ryu C. M. (2015). Gaseous 3-pentanol primes plant immunity against a bacterial speck pathogen, *Pseudomonas syringae* pv. tomato via salicylic acid and jasmonic acid-dependent signaling pathways in *Arabidopsis*. *Front. Plant Sci.* 6:821. 10.3389/fpls.2015.00821 26500665 PMC4593957

[B46] Song G. C. Ryu C. M. (2013). Two volatile organic compounds trigger plant self-defense against a bacterial pathogen and a sucking insect in cucumber under open field conditions. *Int. J. Mol. Sci.* 14 9803–9819. 10.3390/ijms14059803 23698768 PMC3676814

[B47] Speirs J. Lee E. Holt K. Yong-Duk K. Scott N. S. Loveys B. (1998). Genetic manipulation of alcohol dehydrogenase levels in ripening tomato fruit affects the balance of some flavor aldehydes and alcohols. *Plant Physiol.* 117 1047–1058. 10.1104/pp.117.3.1047 9662548 PMC34921

[B48] Spyropoulou E. A. Dekker H. L. Steemers L. van Maarseveen J. H. de Koster C. G. Haring M. A. (2017). Identification and characterization of (3Z):(2E)-hexenal isomerases from cucumber. *Front. Plant Sci.* 8:1342. 10.3389/fpls.2017.01342 28824678 PMC5539243

[B49] Sugimoto K. Matsui K. Takabayashi J. (2015). Conversion of volatile alcohols into their glucosides in *Arabidopsis*. *Commun. Integr. Biol.* 8:e992731. 10.4161/19420889.2014.992731 26629260 PMC4594374

[B50] Tanaka T. Ikeda A. Shiojiri K. Ozawa R. Shiki K. Nagai-Kunihiro N. (2018). Identification of a hexenal reductase that modulates the composition of green leaf volatiles. *Plant Physiol.* 178 552–564. 10.1104/pp.18.00632 30126866 PMC6181032

[B51] van Den Dool H. Kratz P. D. (1963). A generalization of the retention index system including linear temperature programmed gas-liquid partition chromatography. *J. Chromatogr.* 11 463–471. 10.1016/s0021-9673(01)80947-x 14062605

[B52] van Tol R. W. Bruck D. J. Griepink F. C. De Kogel W. J. (2012). Field attraction of the vine weevil *Otiorhynchus sulcatus* to kairomones. *J. Econ. Entomol.* 105 169–175. 10.1603/ec11248 22420269

[B53] Vancanneyt G. Sanz C. Farmaki T. Paneque M. Ortego F. Castañera P. (2001). Hydroperoxide lyase depletion in transgenic potato plants leads to an increase in aphid performance. *Proc. Natl. Acad. Sci. U.S.A.* 98 8139–8144. 10.1073/pnas.141079498 11416166 PMC35481

[B54] Vargas W. A. Martín J. M. S. Rech G. E. Rivera L. P. Benito E. P. Díaz-Mínguez J. M. (2012). Plant defense mechanisms are activated during biotrophic and necrotrophic development of *Colletotricum graminicola* in maize. *Plant Physiol.* 158 1342–1358. 10.1104/pp.111.190397 22247271 PMC3291271

[B55] Vellosillo T. Martínez M. López M. A. Vicente J. Cascón T. Dolan L. (2007). Oxylipins produced by the 9-lipoxygenase pathway in *Arabidopsis* regulate lateral root development and defense responses through a specific signaling cascade. *Plant Cell* 19 831–846. 10.1105/tpc.106.046052 17369372 PMC1867370

[B56] Vicente J. Cascón T. Vicedo B. García-Agustín P. Hamberg M. Castresana C. (2012). Role of 9-lipoxygenase and α-dioxygenase oxylipin pathways as modulators of local and systemic defense. *Mol. Plant* 5 914–928. 10.1093/mp/ssr105 22199234

[B57] Vliegenthart J. F. G. de Groot J. J. M. C. Veldink G. A. Boldingh J. Wever R. Van Gelder B. F. (1975). Demonstration by EPR spectroscopy of the functional role of iron in soybean lipoxygenase-1. *Biochim. Biophys. Acta* 377 71–79. 10.1016/0005-2744(75)90287-9 164225

[B58] Vliegenthart J. F. G. Verhagen J. Bouman A. A. Boldingh J. (1977). Conversion of 9-D-and 13-L-hydroperoxylinoleic acids by soybean lipoxygenase-1 under anaerobic conditions. *Biochim. Biophys. Acta* 486 114–120. 10.1016/0005-2760(77)90075-3 12832

[B59] Vollenweider S. Weber H. Stolz S. Chételat A. Farmer E. E. (2000). Fatty acid ketodienes and fatty acid ketotrienes: Michael addition acceptors that accumulate in wounded and diseased *Arabidopsis* leaves. *Plant J.* 24 467–476. 10.1111/j.1365-313x.2000.00897.x 11115128

[B60] Wang K. D. Borrego E. J. Kenerley C. M. Kolomiets M. V. (2020a). Oxylipins other than jasmonic acid are xylem-resident signals regulating systemic resistance induced by *Trichoderma virens* in maize. *Plant Cell* 32 166–185. 10.1105/tpc.19.00487 31690653 PMC6961617

[B61] Wang K. D. Gorman Z. Huang P. C. Kenerley C. M. Kolomiets M. V. (2020b). *Trichoderma virens* colonization of maize roots triggers rapid accumulation of 12-oxophytodienoate and two ?-ketols in leaves as priming agents of induced systemic resistance. *Plant Signal. Behav.* 15:1792187. 10.1080/15592324.2020.1792187 32657209 PMC8550292

