# Supplementary figures and images for: The Synthesis of Pentyl Leaf Volatiles and Their Role in Resistance to Anthracnose Leaf Blight
Source: Front Plant Sci. 2021 Aug 26;12:719587. doi: 10.3389/fpls.2021.719587 (PMC8427672; doi:10.3389/fpls.2021.719587)

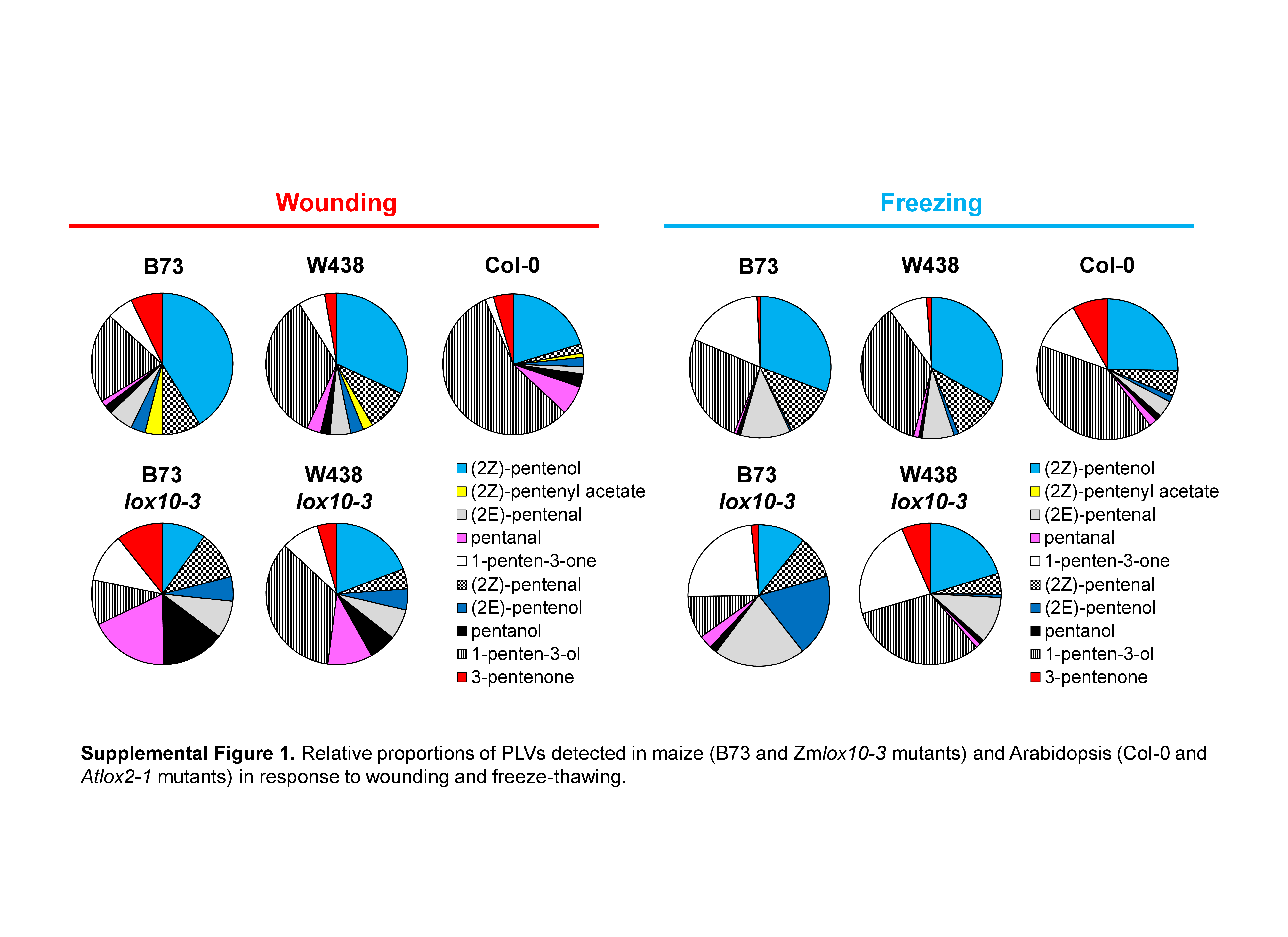

Supplement: Supplementary file 1 [file Image_1.TIFF]

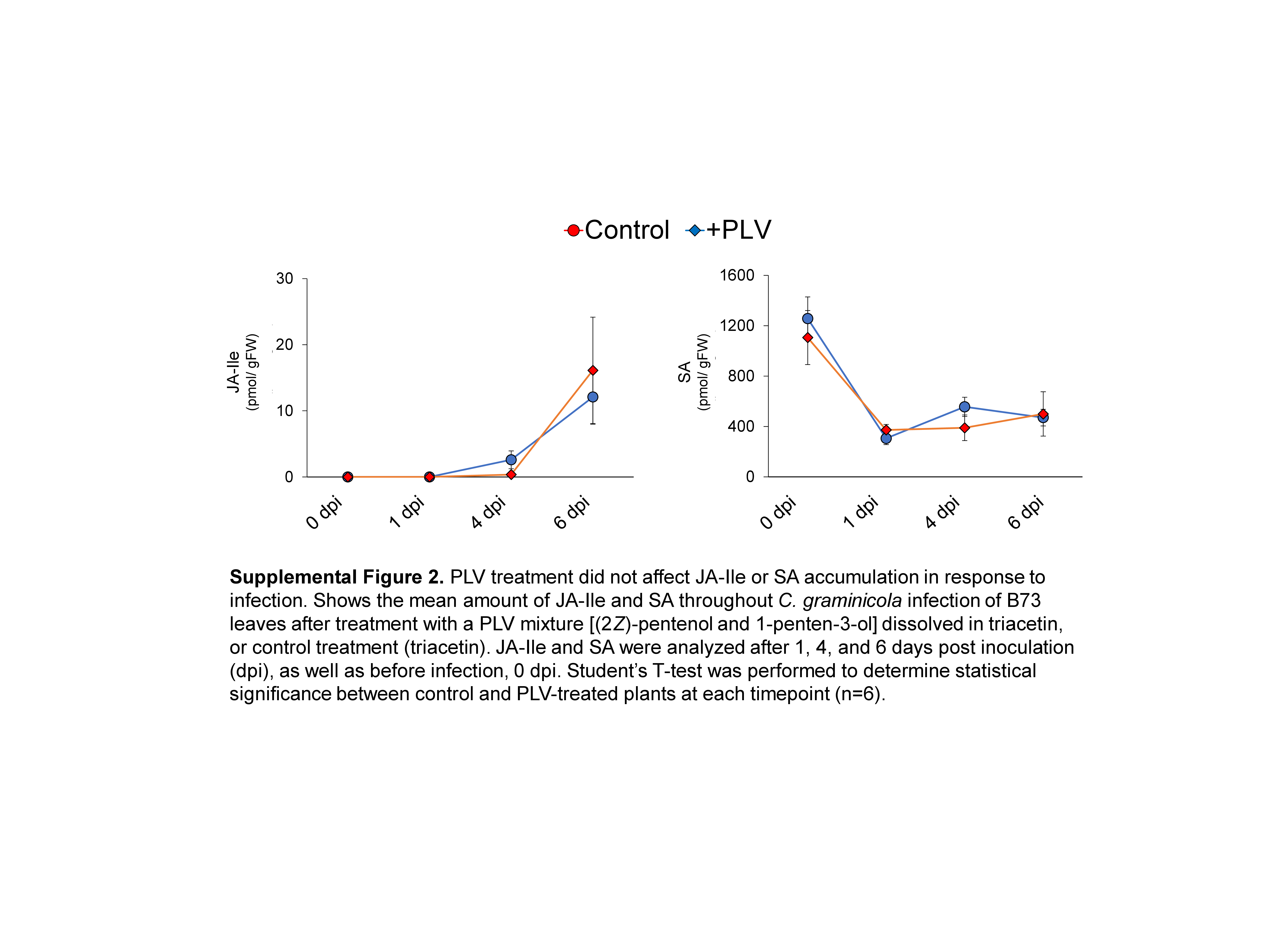

Supplement: Supplementary file 2 [file Image_2.TIFF]
